# Cross-modal interactive and global awareness fusion network for RGB-D salient object detection

**DOI:** 10.1371/journal.pone.0325301

**Published:** 2025-06-12

**Authors:** Runqing Li, Ling Yu, Zijian Jiang, Fanglin Niu

**Affiliations:** School of Electronics and Information Engineering, Liaoning University of Technology, Liaoning, China; Prince Mohammad Bin Fahd University, SAUDI ARABIA

## Abstract

The RGB-D salient object detection technique has garnered significant attention in recent years due to its excellent performance. It outperforms salient object detection methods that rely solely on RGB images by leveraging the geometric morphology and spatial layout information from depth images. However, the existing RGB-D detection model still encounters difficulties in accurately recognising and highlighting salient objects when facing complex scenes containing multiple or small objects. In this study, a Cross-modal Interactive and Global Awareness Fusion Network for RGB-D Salient Object Detection, named CIGNet, is proposed. Specifically, convolutional neural networks (CNNs), which are good at extracting local details, and an attention mechanism, which efficiently integrates global information, are utilized to design two fusion methods for RGB and depth images. One of these methods, the Cross-modal Interaction Fusion Module (CIFM), employs depth separable convolution and common-dimensional dynamic convolution to extract rich edge contours and texture details from low-level features. The Global Awareness Fusion Module (GAFM) is designed to relate high-level features between RGB and depth features so as to improve the model’s understanding of complex scenes. In addition, prediction mapping is generated through a step-by-step decoding process carried out by the Multi-layer Convolutional Fusion Module (MCFM), which gradually yields finer detection results. Finally, comparing 12 mainstream methods on six public benchmark datasets demonstrates superior robustness and accuracy.

## 1 Introduction

The purpose of salient object detection (SOD) is to simulate the human visual system and accurately recognize the most striking objects or regions in various scenes. SOD has been widely used in many computer vision tasks, such as image retrieval [1], object tracking [2], object segmentation [3]and image understanding [4].

With the continuous progress of deep learning, RGB-based saliency object detection gradually overcomes the performance bottleneck of traditional methods and achieves good results. However, when object detection is performed in complex situations such as cluttered backgrounds, coexisting multiple objects, varying illumination, and transparent objects, the results are often unsatisfactory. The core problem lies in the lack of spatial position information, which is crucial for saliency object detection.

In recent years, RGB-D saliency object detection techniques have demonstrated significant performance improvements in complex scenes by introducing depth image information. The image provides rich spatial structure 3D layout information and an accurate object contour definition. However, effectively fusing the information of RGB images and depth images to maximize their respective advantages has been the focus and difficulty of research in this area. Existing RGB-D saliency object detection methods can be broadly categorized into three types: input fusion [5], result fusion [6], and feature fusion [7–9]; however, each method has certain limitations. For example, Chen *et al*. [10]. directly connected three-channel RGB and one-channel depth maps to form a four-channel map. However, directly connecting the two maps leads to ignoring their distributional differences, which ultimately leads to inaccurate feature fusion results. Zhu *et al*. [11]. used a separate subnetwork to extract deep features and integrate these features directly into the RGB network, Fan *et al*. [12]. mined deep information cues by exploring channels and spatial attention mechanisms and subsequently fused this deep information into the RGB features in an auxiliary form. Ben *et al*. [13]. proposed a bio-inspired two-stage network for efficient RGB-D salient object detection, which simulates the visual information processing mechanisms of the P visual pathway and the M visual pathway. Gao *et al*. [14]. proposed a lightweight yet efficient network to address the issues of excessive parameter counts, high computational complexity, and slow inference speeds. Hu *et al*. [15]. proposed a Cross-modal Fusion and Progressive Decoding Network to address the issues of feature redundancy and performance degradation in models. Zhong *et al*. [16]. propose a Multi-scale Awareness and Global Fusion Network to address the challenge of existing detection methods requiring many model parameters to achieve high accuracy. These approaches mainly focus on incorporating the depth features at each level, directly or after enhancement, as auxiliary information into the RGB features and then utilizing a decoder to generate the final saliency maps. However, these fusion strategies do not achieve bidirectional communication between depth features and RGB features; therefore, saliency object detection (SOD) is not effective in the presence of poor quality depth features. Therefore, exploring more efficient and accurate RGB-D SOD fusion strategies to enhance the performance of saliency detection further has become a popular trend in current research.

Current research focuses on feature fusion, which involves constructing two independent networks to extract features from RGB and depth, subsequently methodically integrating these two features and feeding the integrated features into the subsequent model for in-depth learning. Feature fusion enables the model to capture the correlation between features more effectively. One of the primary research challenges is the fusion of feature streams and the complementary advantages of RGB and depth.

Based on the above analysis, this study uses two independent networks, a Scale-aware Modulation Transformer (SMT) and MobileNetV2, to extract RGB features and depth features, respectively, and designed a new RGB-D saliency object detection method based on cross-modal interaction fusion and global awareness fusion. To capture the information interactions present in low-level feature mapping and to reduce the redundancy of features, a Cross-modal Interaction Fusion Module (CIFM) is designed to fuse low-level cross-modal features. To fully utilise the complementary relationship between RGB images and depth images to fuse multimodal useful information on a global scale efficiently, a Global Awareness Fusion Module (GAFM) has been designed. Finally, we constructed a decoder utilising a Multi-layer Convolutional Fusion Module (MCFM) capable of accurately generating saliency maps. The main contributions of this study are summarised as follows.

(1) A Cross-modal Interactive Fusion and Global Awareness Fusion Network (CIGNet) is proposed for RGB-D SOD, featuring a dual-stream encoder-decoder structure. Compared with existing methods, CIGNet has efficient detection performance.

(2) Considering the complementary information relationship between RGB images and depth images, a Global Awareness Fusion Module (GAFM) is designed to fuse abstract high-level semantic information, and a Cross-modal Interaction Fusion Module (CIFM) is proposed, with the abstract high-level semantic information being fused by the Global Awareness Fusion Mdule (GAFM) and the low-level fusion of cross-modal features being performed by the CIFM, enhancing the robustness of the model in low-luminance and complex environments.

(3) A Multi-layer Convolutional Fusion Module (MCFM) is proposed for this study. A decoder capable of accurately generating saliency maps while progressively fusing high-level and low-level features to obtain saliency maps containing rich details from the fused features is constructed by employing a Multi-layer Convolutional Fusion Module.

## 2 Related work

### 2.1 RGB-D salient object detection

Since Itti *et al*. [17]. first proposed the task of salient object detection, many RGB-based salient object detection methods have been proposed with good results. Although RGB-based saliency detection methods perform excellently, it is still challenging to accurately locate salient objects in complex scenes (e.g. cluttered backgrounds and low light). With technological advancements, researchers have begun incorporating depth information into salient object detection. Unlike RGB images, depth images are unaffected by lighting and texture variations and can directly present objects’ spatial structure and contours. Therefore, depth images can effectively compensate for the shortcomings of RGB images when dealing with illumination changes and texture repetition.

In the RGB-D saliency object detection task, RGB features provide rich appearance and texture information, whereas depth features focus on the 3D layout and spatial localisation. Effectively fusing the complementary information of RGB and depth features remains a key challenge in this task. To address this issue, several studies have proposed various solutions. For instance, Zhao *et al*. [18]. designed a coherent disparity aggregation structure to achieve cross-modal and cross-level information fusion through multipath fusion, while Qu *et al*. [19]. utilised hand-designed feature vectors as inputs in combination with a CNN-based model for training, which achieved a significant performance enhancement and demonstrated superior results compared to traditional methods. Piao *et al*. [20]. designed a deep refinement module to fully extract and fuse multi-layer pairwise complementary cues using residual connections to locate salient objects accurately, and Hou *et al*. [21]. aggregated multiscale and multi-layer features in a short-connected manner. Cong *et al*. [22]. combined CNN and transformer architectures to enhance target detection in complex scenes with a cross-modal point perception interaction (CmPI) module and CNN-induced refinement units (CNNR).

In this study, we design cross-modal interaction fusion and global awareness fusion strategies by combining convolutional neural networks and attention mechanisms. Among them, the Cross-modal Interaction Fusion Module (CIFM) focuses on fusing low-level cross-modal features and deeply mining the low-level feature information of the depth and RGB images. The Global Awareness Fusion Module (GAFM) aims to efficiently fuse high-level semantic information between depth and RGB images from a global perspective. The decoder built by the Multi-layer Convolutional Fusion Module (MCFM) combines high-level and low-level feature mappings to generate prediction maps.

### 2.2 RGB-D SOD backbone networks

Since the introduction of convolutional neural networks, RGB-D saliency objective detection (SOD) methods based on CNN technology have emerged. For instance, Zhang *et al*. [23] developed a cross-modal difference interaction strategy to fuse information from different modalities effectively. With the advent of transformers, an increasing number of transformer-based RGB-D SOD models have been proposed. As an example, Liu *et al*. [24]. introduced a pure Transformer architecture for the RGB-D SOD task from a novel perspective of sequence-to-sequence modelling and implemented a cross-attention mechanism in the cross-modal interaction module. Zhou *et al*. [25]et al. utilised the MobileNetV2 network to extract multiscale information from RGB images and provided a new approach for mitigating the potential impact of the lightweight backbone network on the model detection performance; additionally, a boundary enhancement algorithm was developed to prevent information loss in low-dimensional features. Mu *et al*. [26]. conversely, employed the MAVNet n with shared weights to extract multiscale information from RGB images with different viewpoints and investigated the information correlation among RGB images through the multi-view aggregation transformer network.

However, each of the aforementioned backbone networks has unique strengths; CNN is good at capturing localised information, while the transformer is better at dealing with long-distance dependencies. To fully utilise the advantages of both architectures, researchers have started to combine them. For example, Guo *et al*. [27]. proposed the CMT(Convolutional Neural Meet Vision Transformers) network, which combines the local perceptual units of a CNN to extract local relational and structural information. In contrast, the transformer architecture is used to capture global details. Recently, to further enhance the synergistic effect of CNN and Transformer, Lin *et al*. [28]. proposed a Scale-aware Modulation Transformer(SMT) network, which is a new approach that designs an innovative multi-head hybrid convolutional module to capture local information and combines it with transformer architecture to capture global information, which significantly improves the detection performance.

In this study, the Scale-aware Modulation Transformer (SMT) network captures multi-scale information through adaptive scale adjustment. It enhances long-range dependency modelling using the transformer mechanism, thus demonstrating superior accuracy and detail preservation capability when dealing with detail-rich and background-complex RGB images. MobileNetV2, on the other hand, by introducing inverted residuals and linear bottlenecks, can significantly reduce the amount of computation and the number of parameters while still effectively extracting the spatial structure information of the depth map. Therefore, we use an efficient SMT network to extract RGB features and a lightweight MobileNetV2 network to extract depth features.

## 3 Proposed method

**Fig 1:**
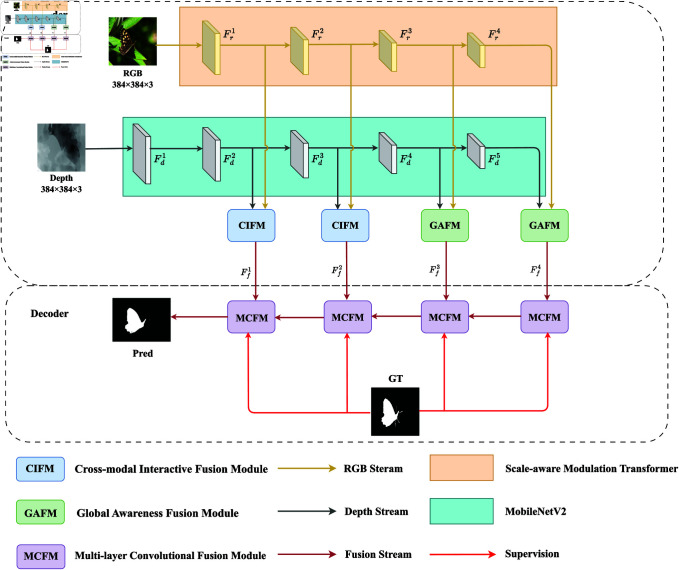
Overall network structure diagram.

A Cross-modal Interactive and Global-aware Fusion Network (CIGNet) is proposed in this paper as shown in Fig 1. CIGNet adopts an encoder-decoder architecture. Specifically, in the RGB branch, the Swin-Aware Modulation Transformer (SMT) is the backbone feature extraction network. In contrast, depth images contain less information than RGB images. We employ MobileNetV2 as the backbone network to extract depth features. Furthermore, given that low-level feature maps retain rich, detailed information and leveraging them can somewhat reduce model complexity, a Cross-modal Interactive Fusion Module (CIFM) is designed to achieve low-level feature fusion. Additionally, since high-level feature maps encapsulate more semantic information with reduced spatial dimensions, a Global Awareness Fusion Module (GAFM) is developed to integrate these features and effectively exploit semantic correlations. Finally, the fused multi-level features are transmitted to a Multi-scale Cascaded Fusion Module (MCFM) to generate high-quality saliency maps.

### 3.1 Encoder

In current research, some scholars employ symmetric Siamese neural network architectures to extract features from RGB visual data and depth information images. However, these two data modalities exhibit significant differences in information representation: RGB images primarily convey surface colour distributions and fine– grained texture features of objects, whereas depth images precisely capture geometric positioning information of targets in three-dimensional space. This study proposes an asymmetric Siamese neural network architecture to address the inherent disparity between these heterogeneous modalities for optimized feature representation extraction.

### 3.2 RGB stream

This paper adopts a hybrid architecture of Scale-aware Modulation Transformer (SMT) as the backbone for RGB image feature extraction. For an input image $I\in R^{H\times W\times 3}$, multi-scale features $F_{r}^{i}$ (i=1,...,4) are obtained through four stages.

### 3.3 Depth stream

Depth images offer crucial three-dimensional cues that enhance foreground-background separation in scenarios with intricate surface patterns. Compared to RGB data that captures chromatic information, depth representations prioritize localized geometric details while containing relatively weaker semantic signals. We employ MobileNetV2 as the core network architecture for processing depth information to balance computational efficiency with feature extraction capabilities. The hierarchical feature maps generated through this depth processing pathway are denoted as $F_{d}^{i}$ (i=1,...,5), capturing spatial characteristics at different scales.

### 3.4 Cross-modal interaction fusion module

**Fig 2:**
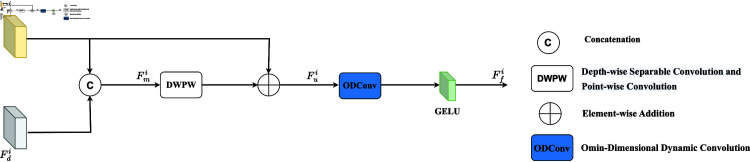
Cross-modal interaction fusion module.

**Fig 3:**
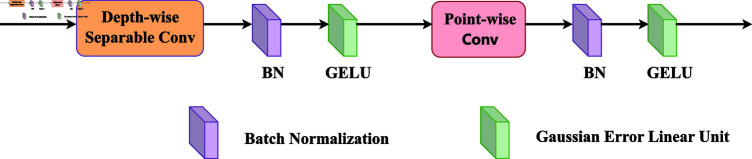
llustration of the DWPW convolution.

In the saliency object detection task, RGB images provide rich scenes and detailed information through a combination of red (R), green (G), and blue (B) channels. In contrast, depth images contain distance data from each pixel point to the camera, which reflects the relative position of the objects in the image and provides more spatial information. To efficiently utilise the complementarity between RGB and depth images, a Cross-modal Interactive Fusion module (CIFM) was designed. As shown in Figs 2 and 3, first, the RGB feature maps and depth feature maps are spliced in the channel dimension, followed by a layer of deep convolution (DW), batch normalisation (BN), and Gaussian error linear units (GeLU). Subsequently, we apply point-by-point convolution (PW), BN layer, and GeLU activation functions to the feature maps to reduce their dimensionality. To avoid information loss, the processed feature maps were then summed with the original RGB feature maps and an Omin-Dimensional Dynamic convolution (ODConv) to enhance the modular feature extraction capability further. Finally, fused feature maps are obtained using the GeLU activation function. The cross-modal interaction fusion is expressed as follows:

$$F_{m}^{i}=Cat\left(F_{r}^{i},F_{d}^{i}\right)$$ (1)

$$F_{u}^{i}=F_{r}^{i}+(\text{DWPW}(F_{m}^{i}))$$ (2)

$$F_{f}^{i}=\sigma (\text{ODConv}(F_{u}^{i}))$$ (3)

Where $F_{r}^{i}$ and $F_{d}^{i}$ (i=1,...,4)denote the RGB and depth features extracted from the backbone network, Cat(·) denotes the connection in the feature map along the channel direction; DWPW(·) denote the depth convolution and point-by-point convolution, respectively; BN and σ denote the normalization layer and GeLU activation function, respectively. ODConv(·) denotes Omin-Dimensional Dynamic convolution.

### 3.5 Global awareness fusion module

**Fig 4:**
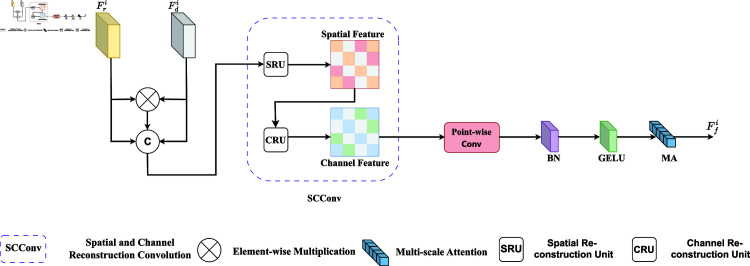
Global awareness fusion module.

Compared to low-level feature maps, high-level feature maps extract more abstract information. Whereas low-level feature maps focus on details and concrete information, high-level feature maps capture semantic information more congruent with human visual intuition and understanding through higher abstraction. We have designed a Global Awareness Fusion Module (GAFM) to address this issue. As illustrated in Fig 4, it aims to fuse the semantic information of different modalities globally. Initially, we multiply the RGB features with the depth features element-by-element at the corresponding locations. We subsequently concatenate this result with the original feature maps in the channel dimension. Subsequently, the fused feature maps are processed by spatial and channel reconstruction convolution (SCConv), point-wise convolution (PW), BN layer, and the GeLU activation function. Following this, an efficient multi-scale attention mechanism (MA) is employed to preserve the information of each channel and reduce the computational overhead by reshaping some of the channels into batch dimensions and grouping the channel dimensions into multiple sub-features. Finally, fused feature mapping was obtained using the GeLU activation function. The global awareness features are fused as follows:

$$F_{f}^{i}=\text{MA}(\sigma (\text{BN}(\text{PW}(\text{SCConv}(\text{Cat}(F_{r}^{i},F_{d}^{i},F_{r}^{i}⊗F_{d}^{i}))))))$$ (4)

Where MA(·) denotes the multiscale attention mechanism, SCConv(·) denotes spatial and channel reconstruction convolution; Cat(·) denotes connectivity in the feature map along the channel direction; PW(·) denotes point-by-point convolution; and BN(·) and σ denote the normalization layer and GELU activation function, respectively.

### 3.6 Multi-layer convolutional fusion module

**Fig 5:**
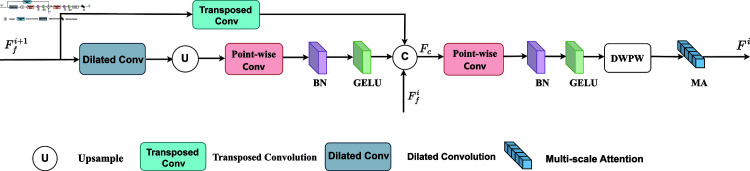
Multi-layer convolutional fusion module.

The decoder constructed by the Multi-layer Convolutional Fusion Module(MCFM) is designed to accept the feature information from the Cross-modal Interaction Fusion Module (CIFM) and the Global Awareness Fusion Module (GAFM). It combines high-level and low-level feature mapping to generate accurate prediction maps step by step. As shown in Fig 5. First, we performed an inverse convolution process on high-level feature mapping to supplement the subsequent feature information. Then, the feature maps are doubled in size by expanding the receptive field and by null convolution and up-sampling operations. Subsequently, we utilized point-by-point convolution to reduce the dimensions of the feature map. Based on this, the processed feature maps are spliced and fused with the next layer’s feature maps and the branching feature maps. Finally, two point-by-point convolution (PW) layers containing batch normalization (BN) and GeLU activation functions, a depth separable convolution (DW) layer containing BN and GeLU and a multi-scale attention mechanism (MA) were used to obtain the final output. The Multi-layer Convolutional Fusion Module is as follows:

$$F_{c}=\text{Cat}(F_{f}^{i},\text{TransConv}(F_{f}^{\left(i+1\right)}),\sigma (\text{BN}(\text{PW}(\text{UP}(\text{Dilated \textasciitilde{}Conv}(F_{f}^{\left(i+1\right)}))))))$$ (5)

$$F^{i}=\text{MA}(\sigma (\text{BN}(\text{PW}(\sigma (\text{BN}(\text{DW}(\sigma (\text{BN}(\text{PW}(F_{c}))))))))))$$ (6)

where UP(·) denotes the upsampling operation,DeConv(·) denotes the inverse convolution operation, and Dilated Conv(·) denotes the null convolution operation. Finally, *F*_*i*_ (i=1,... ,4) uses 1 x 1 convolution to generate the salient map*P*_*i*_(i=1,... ,4) .

### 3.7 Loss function

During the training phase, a hybrid loss composed of binary cross-entropy (BCE) [29]loss and intersection over union (IOU) [30] loss is utilized in this paper to train the network, as shown in Fig 1 effectively. This type of deep supervision improves the accuracy of saliency inference while also quickening the network’s convergence. The BCE loss can be expressed as:

$$L_{BCE}(S,G)=Glog(S,G)+(1-G)log(1-S)$$ (7)

where *G* represents the ground truth, and *S* represents the predicted map.

The IOU loss can be expressed as follows:

$$L_{IOU}(A,B)=1-IOU=1-(\frac{A\cap B}{A\cup B})$$ (8)

where *A* and *B* represent the prediction block diagram and target block diagram, respectively.

The total loss function as L, which can be expressed as:

$$L=L_{BCE}(P^{i},G)+L_{IOU}(P^{i},G)$$ (9)

where *P*^*i*^ denotes the predicted saliency map generated from *F*^*i*^ through a 1×1 convolution operation.

## 4 Experiments

### 4.1 Datasets

This paper mainly carries out experiments on the following six RGB-D datasets covering DUT [31], LFSD [32], NLPR [33], NJU2K [34], STERE [35] and SIP [36]. These datasets are well represented and contain many complex scenes such as similar foreground and background, multiple small objects, and complex backgrounds.

### 4.2 Evaluation metrics

This paper mainly uses six evaluation metrics to evaluate the performance of the model, including Flops, Params , Maximum F-measure($F_{\beta }^{max}$) [37] , Maximum E-measure ($E_{\xi }^{max}$) [38], S-measure (*S*) [39] and mean absolute error (*MAE*) [40]. F-measure ($F_{\beta }$) refers to the weighted harmonic average of recall rate and precision rate under non-negative weight β, and its calculation formula is as follows:

$$F_{\beta }=\frac{(1+\beta^{2})Precision\times Recall}{\beta^{2}Precision+Recall}$$ (10)

From the experience of many salient object detection tasks, $\beta^{2}$ is generally set to a value of 0.3, that is, the weight value of precision is increased. It is believed that the precision rate is more important than the recall rate. E-measure (*E*), which combines the local pixel value with the image-level average value to jointly evaluate the similarity between the predicted value and Ground-truth. The calculation formula of E-measure is:

$$E_{FM}=\frac{1}{w\times h}\sum_{x=1}^{w}\sum_{y=1}^{h}\emptyset_{FM(x,y)},$$ (11)

where *FM* represents the foreground map, ∅ represents an enhanced alignment matrix used to capture the two attributes of binary mapping (pixel-level matching and image-level statistics), *w* and *h* are the width and height of the map, respectively. The S-measure (*S*) focuses on evaluating the structural information of the saliency map, and it is closer to the human visual system than the F-measure. It mainly calculates the structural similarity of object perception and area perception between the predicted value and Ground-truth. The calculation formula of S-measure is:

$$S=\alpha \cdot S_{o}+(1-\alpha )\cdot S_{r},$$ (12)

where *S*_*o*_ and *S*_*r*_ respectively denote the structural similarity of object perception and area perception, and α is generally set to 0.5. Mean Absolute Error (*MAE*) represents the average value of the absolute error between the predicted value and Ground-Truth. The range is [0,+∞). When the predicted value is completely consistent with the GT, it is equal to 0, which is a perfect model; the greater the error, the greater the value. The calculation formula is as follows:

$$MAE=\frac{1}{m}\sum_{i=1}^{m}|y_{i}-f(x_{i})|,$$ (13)

where *m* represents the number of samples, *f*(*x*) represents the predicted value of the model, and *y* represents Ground-truth.

### 4.3 Implementation details

To train and test the model, this uniformly adjusted the input RGB and depth images to a 384 × 384 pixels resolution. To effectively prevent overfitting, a series of enhancement measures are implemented in the data preprocessing stage, including random flipping, cropping, rotating, and colour enhancement. In terms of backbone network parameter initialisation, the pretraining parameters of the SMT and MobileNetV2 networks were adopted. The Adam optimiser was chosen during the model training process, and the batch size was set to 8. The initial learning rate was set to 5e-5, and the learning rate was reduced to one-tenth of the original rate every 80 epochs. The entire training of the model was performed on a server with an NVIDIA RTX 3090 GPU.

### 4.4 Comparative experiment

In this paper, we compare the performance of the model with current representative methods, including HAINet [41], IRFRNet [42], CDNet [43], RD3D [44], M2RNet [45], EGANet [46], HiDANet [47], PICRNet [48], PopNet [49], STANet [50], EMTrans [51]. For a fair comparison with other methods, this paper uses significance plots provided by the authors to compare them qualitatively and quantitatively, respectively.

**Fig 6:**
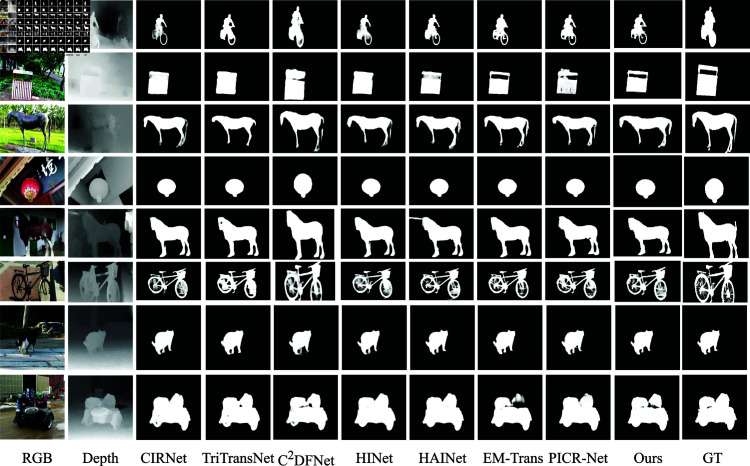
Qualitative comparison of this paper’s algorithm with frontier RGB-D Salient Object Detection models.

(1) Quantitative assessment. The quantitative comparison results between the proposed algorithm and 12 other methods across six datasets are summarized below. As shown in Table 1, our method achieved first-place rankings across all four evaluation metrics on the NLPR dataset, secured top positions in three evaluation metrics for the LFSD dataset, demonstrated superior performance in two metrics each on the NJU2K and SIP datasets, obtained one first-place and three second-place rankings on the STERE dataset, while achieving first place in one evaluation metric for the DUT dataset. These experimental results validate the algorithm’s strong performance in RGB-D salient object detection (SOD) tasks. (2) Qualitative Evaluation. To visually evaluate the algorithms’ performance, we compared the method proposed in this study with a variety of representative state-of-the-art algorithms. A variety of challenging scenarios were purposely selected for comparison, such as highly similar foreground and background (e.g. rows 1 and 3 ), complex and changeable environments (rows 2 and 4), poor quality of the depth map (rows 6 and 7), and the presence of multiple objects (rows 5 and 8). As shown in Fig 6, the model proposed in this study demonstrates higher accuracy in localising and segmenting salient objects and maintains excellent detection capabilities under challenging scenarios. These experimental results further demonstrate the effectiveness and robustness of the proposed model.

**Table 1:** F-Measure ($F_{\beta }^{max}$), MAE (ℳ), S-Measure ($S_{\alpha }$), and E-Measure ($E_{\xi }^{max}$) were compared quantitatively with the RGB-D methods on six widely used RGB-D datasets. The best results are shown in bold black. A "-" symbol represents that the code or result is unavailable.

Data Metric		HAINet	TriTransNet	CIRNet	HINet	C2DFNet	M2RNet	EGANet	HiDANet	PICRNet	PopNet	STANet	EMTrans	Ours
		2021	2021	2022	2022	2022	2023	2023	2023	2023	2023	2024	2024	
FLOP s(G)		181.6	294.0	42.6	389.7	11.1	–	100.7	–	27.1	108.0	85.0	–	37.0
Params(M)		59.8	138.8	103.2	98.9	47.5	172.0	103.8	–	112.0	198.9	24.5	–	26.3
DUT	$E_{\xi }^{max}↑$	0.942	0.964	0.959	–	0.952	0.935	0.880	–	**0.971**	–	–	–	0.967
	ℳ ↓	0.037	0.025	0.029	–	0.031	0.042	0.074	–	**0.021**	–	–	–	**0.021**
	$F_{\beta }^{max}$ ↑	0.917	0.943	0.938	–	0.924	0.925	0.818	–	**0.951**	–	–	–	0.941
	$S_{\alpha }$ ↑	0.910	0.934	0.932	–	0.916	0.903	0.836	–	**0.943**	–	–	–	0.926
NJU2K	$E_{\xi }^{max}↑$	0.940	0.955	0.955	0.945	0.942	0.904	0.949	0.954	0.957	0.952	0.954	0.960	**0.962**
	ℳ ↓	0.039	0.031	0.036	0.039	0.039	0.049	0.035	0.029	0.029	0.030	0.034	0.028	**0.027**
	$F_{\beta }^{max}$ ↑	0.907	0.926	0.927	0.914	0.908	0.922	0.915	**0.939**	0.930	0.936	0.927	0.934	0.935
	$S_{\alpha }$ ↑	0.909	0.921	0.925	0.915	0.907	0.910	0.915	0.926	0.927	0.924	0.924	**0.931**	0.929
NLPR	$E_{\xi }^{max}↑$	0.954	0.965	0.966	0.957	0.961	0.941	0.967	0.961	0.970	–	0.968	0.970	**0.972**
	ℳ ↓	0.026	0.021	0.023	0.026	0.021	0.033	0.021	0.021	0.019	–	0.021	0.018	**0.016**
	$F_{\beta }^{max}$ ↑	0.905	0.922	0.924	0.906	0.915	0.921	0.925	0.929	0.930	–	0.926	0.932	**0.936**
	$S_{\alpha }$ ↑	0.922	0.930	0.933	0.922	0.927	0.918	0.933	0.930	0.936	–	0.934	0.941	**0.942**
STERE	$E_{\xi }^{max}↑$	0.945	0.952	0.924	0.933	0.942	0.929	0.939	0.946	0.955	0.947	0.952	**0.956**	**0.956**
	ℳ ↓	0.037	0.033	0.053	0.049	0.037	0.042	0.041	0.035	0.030	0.033	0.037	**0.028**	0.029
	$F_{\beta }^{max}$ ↑	0.906	0.911	0.896	0.883	0.896	0.913	0.894	0.920	0.918	**0.924**	0.913	**0.924**	0.920
	$S_{\alpha }$ ↑	0.909	0.909	0.888	0.892	0.902	0.899	0.898	0.911	0.921	0.917	0.915	**0.926**	0.923
LFSD	$E_{\xi }^{max}↑$	0.846	0.905	0.908	0.888	0.903	0.874	0.899	–	**0.923**	–	0.905	–	**0.923**
	ℳ ↓	0.105	0.066	0.068	0.076	0.065	0.088	0.069	–	**0.053**	–	0.069	–	**0.053**
	$F_{\beta }^{max}$ ↑	0.778	0.870	0.883	0.847	0.867	0.861	0.865	–	**0.894**	–	0.869	–	0.887
	$S_{\alpha }$ ↑	0.796	0.867	0.875	0.852	0.863	0.842	0.861	–	0.888	–	0.867	–	**0.891**
SIP	$E_{\xi }^{max}↑$	0.927	0.930	0.919	0.899	0.912	0.921	0.924	0.927	0.939	0.937	0.943	0.943	**0.945**
	ℳ ↓	0.049	0.044	0.056	0.066	0.056	0.049	0.049	0.043	0.041	0.040	0.040	0.040	**0.036**
	$F_{\beta }^{max}$ ↑	0.901	0.899	0.890	0.855	0.871	0.902	0.891	0.919	0.912	**0.923**	0.919	0.920	0.921
	$S_{\alpha }$ ↑	0.866	0.887	0.881	0.856	0.867	0.882	0.883	0.892	0.896	0.897	0.902	**0.907**	0.906

### 4.5 Future work

Although the experimental results demonstrated by CIGNet have proven the effectiveness and practicality of the proposed method, there are still certain limitations in this study’s lightweight problem. The model has many parameters and high computational complexity, resulting in low deployment efficiency on edge devices and making it difficult to meet the requirements of real-time application scenarios. Therefore, future work should focus on the design of lightweight architectures to address these challenges.

## 5 Ablation analysis

In this section, we conducted ablation experiments on the LFSD, NLPR, and NJU2K datasets to evaluate the contributions of key components in the proposed method. As shown in Table 2, ablation experiments were carried out to assess the effectiveness of each module in CIGNet.In case (a), when the cross-modal interaction fusion module (CIFM) was removed, and only the high-level semantic information of RGB images and depth images was processed and fused, the model’s performance showed a significant decline. In case (b), when the global attention fusion module (GAFM) was removed, and only the low-level semantic information of RGB images and depth images was processed and fused, a notable deterioration in the model’s performance was also triggered. However, the effect was slightly better than that in case (a). It can be inferred that the cross-modal interaction fusion module (CIFM) plays a central role in effectively fusing the high-level semantics of RGB images and depth images and maintaining the model’s performance. In contrast, the global attention fusion module (GAFM) serves an auxiliary optimization function in establishing correlations for low-level semantics. The two modules are complementary at the feature level, with CIFM being the key element. In case (c), when the MCFM module was removed, and a single-layer convolution was used as the decoder to fuse the feature information from the encoder, the performance dropped significantly. This can be attributed to the fact that a single-layer convolution struggles to achieve cross-layer interaction and semantic integration of the multi-scale features from the encoder. In contrast, the MCFM ensures in-depth feature integration through its structured fusion mechanism. The absence of MCFM leads to a significant reduction in the model’s representation capability.

**Table 2:** Quantitative results of the cross-modal interaction fusion module. (The best results are shown in bold black).

No.		NLPR				LFSD				NJU2K			
		$E_{\xi }^{max}↑$	ℳ↓	$F_{\beta }^{max}↑$	$S_{\alpha }↑$	$E_{\xi }^{max}↑$	ℳ↓	$F_{\beta }^{max}↑$	$S_{\alpha }↑$	$E_{\xi }^{max}↑$	ℳ↓	$F_{\beta }^{max}↑$	$S_{\alpha }↑$
*a*	w/oCIFM	0.914	0.061	0.879	0.880	0.969	0.018	0.930	0.937	0.958	0.029	0.927	0.925
*b*	w/oGAFM	0.918	0.056	0.883	0.885	0.970	**0.016**	0.931	0.939	0.959	**0.027**	0.932	0.928
*c*	w/oMCFM	0.919	0.056	0.885	0.884	0.968	0.019	0.921	0.931	0.953	0.033	0.919	0.917
*d*	Ours	**0.922**	**0.053**	**0.887**	**0.891**	**0.972**	**0.016**	**0.936**	**0.942**	**0.962**	**0.027**	**0.935**	**0.929**

### 5.1 Effectiveness of cross-modal interaction fusion module

Three experiments were conducted to validate the effectiveness of the Cross-modal Interaction Fusion Module. In case (a), the removal of the CIFM is performed using element-based simple additive RGB feature mapping and depth feature mapping as a baseline model. In case (b), the cross-modal fusion module (CMFM) of the CATNet [52]was used. In case (c), the Cross-modal Interactive Fusion module (CIFM) proposed in this paper is used. The experimental results are listed in Table 3. According to the experimental results in Table 3, after adding the CIFM to the baseline model, our proposed model achieved significant performance improvements in all four evaluation metrics on the three datasets. The results show that our designed CIFM can flexibly consider the relationship between features and the relationship between other related features in context, which makes the feature fusion more comprehensive.

**Table 3:** Quantitative results of the global awareness fusion module. (The best results are shown in bold black).

No.		NLPR				LFSD				NJU2K			
		$E_{\xi }^{max}↑$	ℳ↓	$F_{\beta }^{max}↑$	$S_{\alpha }↑$	$E_{\xi }^{max}↑$	ℳ↓	$F_{\beta }^{max}↑$	$S_{\alpha }↑$	$E_{\xi }^{max}↑$	ℳ↓	$F_{\beta }^{max}↑$	$S_{\alpha }↑$
*a*	*Baseline*	0.914	0.061	0.879	0.880	0.969	0.018	0.930	0.937	0.958	0.029	0.927	0.925
*b*	w/oCIFM+CMFM	0.911	0.058	0.880	0.878	0.970	0.017	0.932	0.938	0.960	0.028	**0.936**	0.927
*c*	Ours	**0.922**	**0.053**	**0.887**	**0.891**	**0.972**	**0.016**	**0.936**	**0.942**	**0.962**	**0.027**	**0.935**	**0.929**

### 5.2 Effectiveness of the global awareness fusion module

To validate the effectiveness of the GAFM, we designed the following experiments. In case (a), GAFM is removed using element-based simple additive RGB feature mapping and depth feature mapping as a baseline model. In case (b), we utilise the Attention Fusion (AF) module of RFNet [53]. In case (c), the Global Awareness Fusion Module (GAFM) proposed in this study is used. The experimental results are listed in Table 4. The experimental results in Table 4, compared with the baseline model (a), suggest that our GAFM can effectively capture the complex correspondence between the RGB and depth features, thus facilitating deeper fusion.

**Table 4:** Quantitative results of the global awareness fusion module. (The best results are shown in bold black).

No.		NLPR				LFSD				NJU2K			
		$E_{\xi }^{max}↑$	ℳ↓	$F_{\beta }^{max}↑$	$S_{\alpha }↑$	$E_{\xi }^{max}↑$	ℳ↓	$F_{\beta }^{max}↑$	$S_{\alpha }↑$	$E_{\xi }^{max}↑$	ℳ↓	$F_{\beta }^{max}↑$	$S_{\alpha }↑$
*a*	*Baseline*	0.918	0.056	0.883	0.885	0.970	**0.016**	0.931	0.939	0.959	**0.027**	0.932	0.928
*b*	w/oGAFM+AF	0.919	0.057	0.883	0.884	0.969	0.017	0.933	0.939	0.961	0.028	0.933	0.928
*c*	Ours	**0.922**	**0.053**	**0.887**	**0.891**	**0.972**	**0.016**	**0.936**	**0.942**	**0.962**	**0.027**	**0.935**	**0.929**

### 5.3 Effectiveness of multi-layer convolutional fusion module

To verify the effectiveness of the MCFM.In case (a), the baseline model replaces the multi-layer fusion decoder with a single-layer convolutional decoder. In case (b), the low-level feature map fusion is removed, denoted as MCFM′. In case (c), the proposed MCFM is fully adopted. As shown in Table 5, MCFM demonstrates superior detection performance compared to the baseline model (a). This improvement stems from the residual structure of MCFM, which incorporates multiple convolutional blocks rather than a single-layer convolution, thereby providing enhanced representational capacity. Consequently, MCFM better captures high-level semantic features and structural patterns in images and achieves more effective feature map decoding. When compared to MCFM’ (b) without low-level feature fusion, MCFM exhibits further performance gains. This advantage arises because MCFM fully exploits multi-scale feature map information, empowering the decoder with stronger learning capabilities and improved generalization.

## 6 Conclusion

This study focuses on RGB-D salient object detection to effectively integrate local and global information through feature fusion. We adopted the SMT network and MobileNetV2 network to extract RGB and depth features, respectively, to utilise feature extraction’s advantages fully. This study innovatively introduces a Cross-modal Interaction Fusion Module(CIFM) and a Global Awareness Fusion Module(GAFM) to enhance the feature fusion effect. The former fuses low-level features to capture subtle changes in the image, and the latter fuses high-level features to understand image objects and scenes accurately. Together, these two modules realise the multidimensional and multilevel fusion of RGB and depth features. In addition, a decoder containing a Multi-layer Convolutional Fusion Module(MCFM) is designed to refine and enhance the features output from the encoder so that the model focuses more on key details and extracts more delicate and accurate feature information. We conducted comprehensive experiments on six publicly available benchmark datasets to validate the proposed module’s effectiveness and the model’s performance. The experimental results show that the model in this study performs well in the salient object detection task, demonstrating the proposed method’s effectiveness and practicality.

**Table 5:** Quantitative results of the multi-layer convolutional fusion module. (The best results are shown in bold black).

No.		NLPR				LFSD				NJU2K			
		$E_{\xi }^{max}↑$	ℳ↓	$F_{\beta }^{max}↑$	$S_{\alpha }↑$	$E_{\xi }^{max}↑$	ℳ↓	$F_{\beta }^{max}↑$	$S_{\alpha }↑$	$E_{\xi }^{max}↑$	ℳ↓	$F_{\beta }^{max}↑$	$S_{\alpha }↑$
*a*	*Baseline*	0.919	0.056	0.885	0.884	0.968	0.019	0.921	0.931	0.953	0.033	0.919	0.917
*b*	MCFM′	0.917	0.054	0.885	0.886	0.966	0.018	0.925	0.929	0.950	0.029	0.922	0.925
*c*	Ours	**0.922**	**0.053**	**0.887**	**0.891**	**0.972**	**0.016**	**0.936**	**0.942**	**0.962**	**0.027**	**0.935**	**0.929**
